# *Pichia pastoris*-Derived β-Glucan Capsules as a Delivery System for DNA Vaccines

**DOI:** 10.3390/vaccines12121428

**Published:** 2024-12-18

**Authors:** Samara Sousa de Pinho, Maria da Conceição Viana Invenção, Anna Jéssica Duarte Silva, Larissa Silva de Macêdo, Benigno Cristofer Flores Espinoza, Lígia Rosa Sales Leal, Marco Antonio Turiah Machado da Gama, Ingrid Andrêssa de Moura, Micaela Evellin dos Santos Silva, Débora Vitória Santos de Souza, Marina Linhares Lara, Julia Nayane Soares Azevedo Alves, Antonio Carlos de Freitas

**Affiliations:** Laboratory of Molecular Studies and Experimental Therapy—LEMTE, Department of Genetics, Federal University of Pernambuco, Recife 50670-901, Brazil; samara.pinho@ufpe.br (S.S.d.P.); maria.conceicao@ufpe.br (M.d.C.V.I.); anna.jessica@ufpe.br (A.J.D.S.); larissa.smacedo@ufpe.br (L.S.d.M.); benigno.cristofer@ufpe.br (B.C.F.E.); ligia.leal@ufpe.br (L.R.S.L.); marco.turiah@ufpe.br (M.A.T.M.d.G.); ingrid.andressa@ufpe.br (I.A.d.M.); micaela.evellin@ufpe.br (M.E.d.S.S.); debora.vsouza@ufpe.br (D.V.S.d.S.); marina.linharesl@ufpe.br (M.L.L.); julia.nayane@ufpe.br (J.N.S.A.A.)

**Keywords:** glucan particle, yeast cell wall, nucleic acids

## Abstract

Background/Objectives: DNA vaccines are rapidly produced and adaptable to different pathogens, but they face considerable challenges regarding stability and delivery to the cellular target. Thus, effective delivery methods are essential for the success of these vaccines. Here, we evaluated the efficacy of capsules derived from the cell wall of the yeast *Pichia pastoris* as a delivery system for DNA vaccines. Methods: The capsules were extracted from the yeast *Pichia pastoris* strain GS115, previously grown in a YPD medium. pVAX1 expression vector was adopted to evaluate the DNA vaccine insertion and delivery. Three encapsulation protocols were tested to identify the most effective in internalizing the plasmid. The presence of plasmids inside the capsules was confirmed by fluorescence microscopy, and the encapsulation efficiency was calculated by the difference between the initial concentration of DNA used for insertion and the concentration of unencapsulated DNA contained in the supernatant. The capsules were subjected to different temperatures to evaluate their thermostability and were co-cultured with macrophages for phagocytosis analysis. HEK-293T cells were adopted to assess the cytotoxicity levels by MTT assay. Results: The microscopy results indicated that the macrophages successfully phagocytosed the capsules. Among the protocols tested for encapsulation, the one with 2% polyethylenimine for internalization showed the highest efficiency, with an encapsulation rate above 80%. However, the vaccine capsules obtained with the protocol that used 5% NaCl showed better thermal stability and encapsulation efficiency above 63% without induction of cell viability loss in HEK 293T. Conclusions: We successfully described a vaccine delivery system using yeast capsules derived from *Pichia pastoris*, demonstrating its potential for DNA vaccine delivery for the first time. Additional studies will be needed to characterize and improve this delivery strategy.

## 1. Introduction

Vaccination is one of the most effective health interventions for preventing infectious diseases. However, the emergence of pathogens and viral mutations has highlighted the need for new vaccine platforms [1,2]. Although effective, conventional vaccines, such as attenuated, inactivated, and protein subunit vaccines, face limitations regarding scope, development time, and immune response. In this context, innovation in vaccine platforms is essential to meet health demands, especially in outbreak and pandemic situations [3]. Nucleic acid vaccines, such as those based on DNA and RNA, have emerged as a promising alternative being approved for use in humans since the COVID-19 pandemic with three vaccines already licensed: Spikevax (Moderna), Comirnaty (Pfizer-BioNTech), and ZyCoV-D (Cadila). These licenses open doors to new possibilities of approved DNA-based vaccines, as they reinforce the efficacy of this vaccine platform that offers rapid development and flexibility in adapting to different pathogens. However, these vaccines still face challenges, such as the instability of the genetic material and the efficiency of delivery to the cellular target [4]. Overcoming these limitations is crucial to maximize the potential of these new approaches.

A key aspect of the success of nucleic acid vaccines lies in the choice of appropriate delivery systems that can guarantee antigen stability and promote efficient capture by antigen-presenting cells (APCs) [5,6]. In this sense, several delivery systems have been developed to maximize the efficacy of these vaccines, such as delivery systems based on lipids, polymeric structures, and recombinant microorganisms [7,8,9].

Yeasts have gained recognition as an innovative and promising delivery system for nucleic acid vaccines [10]. Species such as *Pichia pastoris* and *Saccharomyces cerevisiae* are widely employed in biotechnology due to their safety, rapid growth, and ease of genetic manipulation [11]. Yeast-based technology offers a potentially safer and more stable alternative, with a lower risk of toxicity and adverse reactions than lipid systems and viral vectors [12]. These organisms can be explored in another presentation form, based on the composition of the yeast cell wall to generate capsules, also named yeast shells (YS) [13,14]. This system takes advantage of the natural robustness of the cell wall to encapsulate and protect the genetic material or antigens, facilitating controlled delivery and effective interaction with the immune system [15].

Yeast capsules have advantages in storage and transportation. Unlike many lipid systems or viral vectors that require extremely low-temperature storage conditions, yeast capsules are stable over a wider range of conditions, making them ideal for distribution in areas with limited infrastructure. This stability, combined with the ease of large-scale production, makes this technology highly attractive for mass vaccination campaigns, especially in outbreak or pandemic scenarios [15,16,17].

Additionally, yeast capsules demonstrate superior delivery efficiency, allowing for the controlled release of antigens and enhancing the immune response. This delivery capability is crucial to maximizing the efficacy of vaccines by ensuring the suitable antigen presentation [18]. Additionally, yeast capsules have the potential for an oral route of administration that can increase public acceptance and improve treatment adherence owing to their non-invasive nature and ease of use [18,19,20]. This method not only simplifies the delivery process but also provides notable immunological advantages [21]. Oral administration effectively mimics the natural route of infection for various pathogens, thereby stimulating a robust mucosal immune response [21,22]. This response includes the production of secretory IgA antibodies and the activation of gut-associated lymphoid tissues (GALT), which are crucial for combating pathogens and promoting both local and systemic immunity [21,23]. The possibility of using them to carry antigens also opens up new perspectives for immunization against infections and the development of therapeutic vaccines and immunotherapies against cancer-targeting components of the tumor microenvironment as tumor-associated macrophages [4,24,25,26]. Furthermore, the B-glucans present in yeast derivatives interact with Dectin-1 receptors of antigen-presenting cells (APCs), which enables the phagocytosis of this vaccine antigen carried by yeast capsules, overcoming the limitation of naked DNA vaccines, which usually present difficulties in this transport and presentation to immune system cells [27,28].

To date, most studies have focused on the use of *S. cerevisiae*, whose structural and physiological characteristics are well understood, paving the way for significant advances in vaccine application and also in exploring the potential of other yeast species [18]. In this sense, we herein present the development of capsules derived from the cell wall of the yeast *P. pastoris* as a model antigen delivery system. We aimed to present the characterization of this delivery platform as the primary objective of the present study, focusing on its thermostability, phagocytic capacity, cytotoxicity, surface charges, and capacity to internalize DNA without the possibility of degradation, thus demonstrating its potential application as a delivery vehicle for DNA-based vaccines. Thus, this capsule-based transport system has the potential to be improved as a new type of vaccine vehicle, expanding the options available for vaccination strategies.

## 2. Materials and Methods

### 2.1. Plasmid DNA

The pVAX1 expression vector (Invitrogen, Carlsbad, CA, USA) was employed to evaluate the insertion and delivery of DNA vaccines. Plasmid DNA was propagated in *Escherichia coli* Top10 [F- *mcrA* Δ (*mrr-hsd*RMS-*mrc*BC) Ø80*lac*ZΔM15 Δ*lac*X74 *rec*A1 *deo*R *ara*D139 Δ(*ara-leu*)7697 *gal*U *gal*K *rsp*L (StrR) *end*A1 *nup*G] (Invitrogen, Carlsbad, CA, USA). Transformed cells were cultured and selected in LB medium supplemented with 50 μg/mL kanamycin.

### 2.2. Yeast Cultivation

*P. pastoris* GS115 yeast cells (Genotype: *His4* and Phenotype: Mut^+^ and His-) were cultured in 1 L of liquid YPD medium (1% yeast extract, 2% tryptone, and 2% glucose) in an aerated flask in a shaking incubator at 150 rpm at 30 °C for 48 h. The cells were collected by centrifugation at 2000× *g* for 5 min, washed three times in sterile distilled water, and had their weight measured.

### 2.3. Obtaining the Capsules

**Figure 1:**
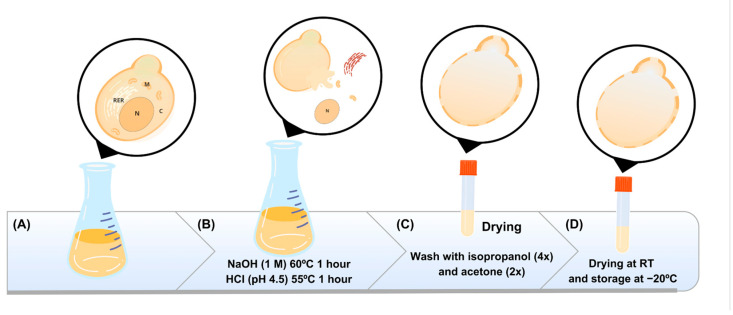
Schematic representation of the methodology used for capsule preparation. This figure outlines the process of yeast cell treatment and preparation. In (**A**), the whole yeast cells are cultured, providing the starting material for the yeast capsule procedure; after that, the cells undergo a chemical treatment (**B**), first with NaOH (1 M) at 60 °C for 1 h to disrupt cell components, followed by HCl (pH 4.5) at 55 °C for 1 h to further process and clean the cellular material. The treated material is subjected to multiple washes (**C**), including four rounds with isopropanol and two with acetone, to remove impurities and ensure purity of the capsules. The final capsules are dried at room temperature (RT) and stored at −20 °C to maintain their integrity and stability for further applications (**D**).

The protocol by Zhu et al. (2021) was adapted to obtain capsules composed of the cell wall of *P. pastoris* GS115 [29]. The yeast biomass obtained after cultivation was suspended in 160 mL of NaOH (1.0 M) and incubated for 1 h at 60 °C under agitation. Then, the mass was centrifuged at 2000× *g* for 10 min. The precipitate was then suspended in 160 mL of water (pH 4.5, adjusted with HCl) and incubated at 55 °C for 1 h. The capsules present in the precipitate were centrifuged and washed once with 70 mL of water. Then, the capsules were washed with 32 mL of isopropanol (4 times) and with 32 mL of acetone (twice) (Figure 1). The precipitate was dried at room temperature and stored at −20 °C until use. The formation of these particles was monitored using optical microscopy, as well as the seeding of the capsules on YPD plates to verify reproductive inactivity, and incubated for 48 h at 30 °C. To evaluate morphological differences or changes in *P. pastoris* yeasts before and after capsule preparation, cells and capsules were stained following the protocol proposed by Kwolek-Mirek e Zadrag-Tecza (2014) [30]. Briefly, a drop of biological material (in PBS 1x) was placed on the slide, followed by a drop of methylene blue. Then, the preparation was covered with a coverslip for observation under an ^®^ K55 OIT optical microscope (Kasvi, Pinhais, PR, Brazil), using magnifications of 40× and 100×. The obtained capsules, also named yeast shells (YS), were quantified using the method described by Huang et al. (2010), where the particles were suspended in 0.9% saline solution and counted in a hemocytometer (5 quadrants) [31]. The particles were kept at −20 °C until use.

### 2.4. Internalization of Plasmids into Capsules

**Figure 2:**
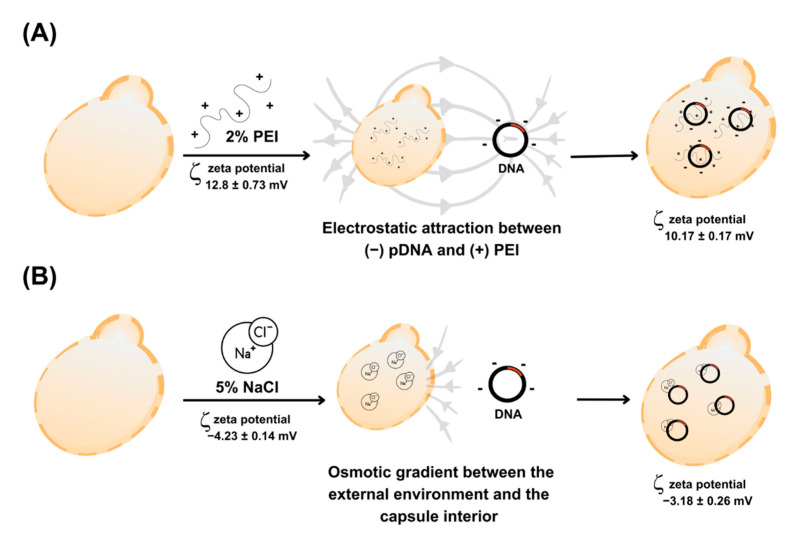
Methodologies for inserting plasmid DNA into the capsules. (**A**) Insertion methodology 1 (PI-1) using 2% PEI; (**B**) insertion methodology 2 (PI-2) using 5% NaCl. Grey arrows represents eletrostatic interactions between DNA and PEI/NaCl.

Three protocols were performed to insert plasmid DNA into the capsules aiming to choose the one with the best encapsulation efficiency (EE%). For all protocols, 1 × 107 capsules were resuspended in 1 mL of PBS for further DNA encapsulation, and three DNA concentrations were tested (50, 250, and 500 ng/μL). In all experiments, the quantification of the capsules was determined using the Countess 3 Automated Cell Counter (Invitrogen, Carlsbad, CA, USA), used to count particles with a diameter of ~4–60 µm. The insertion protocol 1 (PI-1) was based on a method described by Ostroff et al. (2005), with modifications [13]. An aliquot of 25 μL of capsule preparation was mixed with 200 μL of 2% branched polyethylenimine (PEI) solution (Mw ~25,000 by LS; Sigma-Aldrich, St. Louis, MI, USA) and 25 μL of plasmid DNA (Figure 2A). The samples remained for 30 min at room temperature (RT). Then, 75 μL of 95% ethanol was added and incubated at 20 °C for 16 h. The solution was centrifuged at 2000× *g* for 10 min, washed with 70% ethanol, and centrifuged in the same conditions. Finally, the supernatant was discarded, and the capsules with internalized DNA were stored at −20 °C until use. For the insertion protocol 2 (PI-2), the method described by Paramera et al. (2011) was adopted, with some adaptations [32]. Then, 25 μL of capsules were mixed with 25 μL of 5% NaCl and 25 μL of DNA (Figure 2B) and incubated at 55 °C, under agitation of 150 rpm for 48 h, with subsequent centrifugation at 2000× *g* for 10 min, washing with 70% ethanol and centrifuged. The resulting capsules carrying the DNA were stored at −20 °C until use. In insertion protocol 3 (PI-3), the capsules were prepared without anionic solutions. Thus, 25 μL of capsules were added to 25 μL of DNA. The capsules were incubated at 55 °C, under 150 rpm agitation, for 48 h. Subsequently, the solution was centrifuged at 2000× *g* for 10 min and washed with 70% ethanol. The resulting capsules were stored at −20 °C until use.

### 2.5. Fluorescence Microscopy

The presence of plasmid DNA inside the capsules was verified by fluorescence microscopy following the method described by Paramera et al. (2010), with modifications [32]. The 1 × 10^7^ capsules, containing approximately 250 ng/µL DNA, were homogenized in 10 μL of distilled water and placed on slides. Afterward, 5 μL of 4′,6-diamidino-2-phenylindole (DAPI) at a final concentration of 150 ng/mL were added to the slides to allow blue fluorescent labeling when bound to the DNA and washed to remove dye excess. They were then covered with a coverslip. A negative control group (empty capsules) and samples of capsules containing the construction with the pVAX1 vector (2% PEI and 5% NaCl) were used.

### 2.6. Encapsulation Efficiency (EE%)

To verify how much DNA was internalized in the capsules, the encapsulation efficiency was calculated. This index reflects the difference between the initial concentration of inserted DNA (IC) and the final concentration measured from the supernatant corresponding to the unencapsulated DNA (UED). DNA measurement was performed by spectrophotometry using NanoDrop (Thermo Scientific, Waltham, MA, USA). The encapsulation efficiency calculation, in percentage, was defined from the following equation: (% EE) = [(IC) − (UED)/(IC)] × 100.

### 2.7. Thermal Stability Test

The thermostability of the capsules containing the DNA was evaluated following the protocol proposed by Shi et al. (2007), with modifications [33]. The samples were incubated for 11 days at different temperatures: 8 °C, 26 °C (room temperature), and 65 °C. After this period, the encapsulation efficiency (EE%) and DNA retention after heat treatment were analyzed. The samples were subjected to these temperature variations to evaluate the ability of the capsules to retain the DNA, as well as to determine the best storage conditions. The lost DNA was measured from the supernatant of the preparations using NanoDrop (Thermo Scientific, Waltham, MA, USA), in triplicate. The retained DNA is the result of subtracting the incorporated and lost DNA.

### 2.8. Phagocytosis Analysis

To visualize internalization in macrophages, the capsules were stained with 5′-DTAF (3-5-(4,6-dichlorotriazinyl)aminofluorescein) (Invitrogen, Carlsbad, CA, USA), according to the method described by Aouadi et al. (2009), with modifications [34]. Thus, 1 × 10^7^ capsules were washed with 0.1 M sodium carbonate buffer (pH 9.2) and resuspended in 0.1 L of the buffer. 5′-DTAF was added to the suspension of buffered capsules (10% *v*/*v*), and the mixture was incubated at 20 °C in the dark for 16 h. Then, the capsules were incubated in a 2 mM Tris buffer for 15 min and washed with distilled water to remove excess dye. Finally, the labeled capsules were dehydrated with absolute ethanol and acetone, dried in the dark at 20 °C, and stored at −20 °C until use. The macrophages used for phagocytosis were derived from THP-1 monocytes. For this purpose, the cells were cultured in DMEM medium (Sigma-Aldrich, St. Louis, MI, USA) supplemented with 10% (*v*/*v*) inactivated Fetal Bovine Serum (Sigma-Aldrich, St. Louis, MI, USA) and 1% (*v*/*v*) Penicillin–Streptomycin (Thermo Scientific, Waltham, MA, USA) and maintained in a humid incubator with 5% CO_2_ at 37 °C. For differentiation into macrophages, THP-1 cells were seeded at a density of 1 × 10^5^/well in an 8-well Nunc^®^ Lab-Tek^®^ II Chamber Slide™ system (Sigma-Aldrich, St. Louis, MI, USA) and incubated in supplemented DMEM medium containing 150 nM PMA (Phorbol 12-Myristate 13-Acetate; Sigma-Aldrich, St. Louis, MI, USA) for 24 h. After this period, the culture medium was exchanged for supplemented DMEM medium, and the cells were incubated for an additional 24 h. After this period, the culture medium was removed to wash the cells with PBS pH 7.4 twice, and fresh supplemented DMEM medium was added. The MØ macrophages were incubated with 1 × 10^7^ stained capsules for 4 h. Prior to visualization by microscopy, macrophages were stained with DAPI, and the cells were washed to remove any free YS, ensuring that the observed fluorescence corresponds to phagocytosed YS rather than extracellular particles. This experiment was performed in triplicate. Phagocytosis was evaluated by fluorescence microscopy using a Motic AE31E microscope, and the images were captured by a Moticam S6 camera (Universal City, TX, USA) using Motic Images Plus 3.0 software.

### 2.9. Cytotoxicity Assessment

For this assay, HEK-293T cells were used, following the protocol described by Monsmann (1983) with adaptations [35]. Briefly, HEK-293T cells were centrifuged, counted, and seeded (1 × 10^5^ cells/well) in 96-well plates containing 100 μL of DMEM medium supplemented with 10% heat-inactivated FBS. The cells were adhered overnight at 37 °C and 5% CO_2_. Then, the culture supernatant was removed, and 100 μL of the samples, diluted in DMEM, were added to the cells. The cells were incubated with empty capsules and capsules with DNA (approximately 250 ng/μL) incorporated with 2% PEI and 5% NaCl, constituting three experimental groups. The concentration of yeast shells was 5×10^4^/well. The cells were maintained for 24 h. After the incubation period, the supernatant was removed, and the cells adhered to the well were incubated with 5 mg/mL of 3-(4,5-dimethylthiazol-2-yl)-2,5-diphenyltetrazolium bromide (MTT, Sigma-Aldrich) diluted in DMEM, maintained for 4 h at 37 °C and 5% CO_2_. After incubation, the supernatant was discarded, and the cells were solubilized in 100 μL of DMSO to resuspend the formazan crystals. Absorbance reading was performed using a GloMax^®^ Discover Microplate Reader (Promega, Madison, WI, USA) at a wavelength of 570 nm. Absorbance values were transformed into percentage of cell viability, based on the mean absorbance of the negative control (untreated HEK 293-T cells) using the GraphPad Prism^®^ version 9.0 program. Tests were performed in 6 biological replicates.

### 2.10. Zeta Potential (ζ) Measurements

The ζ analysis was performed on empty capsules treated with PEI 2% and NaCl 5% (controls), as well as DNA-loaded capsules treated under the same conditions. Briefly, 1 × 10^7^ capsules from each sample were prepared and adjusted to a final dilution of 1:10 in PBS 1x. The ζ measurements were conducted in triplicate using the Zetasizer Nano ZS90 instrument (Malvern, WR, United Kingdom), employing the dynamic light scattering technique. The data obtained were processed using Technology Software 7.01. Prior to the measurements, all samples were washed with ultra-pure water. Analyses were assessed at room temperature and a pH of approximately 7.0.

### 2.11. DNAse Assay

The capsules with internalized plasmid DNA were tested for their protective capacity in the presence of DNase. For this purpose, a DNase I assay (Thermo Scientific, Waltham, MA, USA) was performed. The reactions were assembled following the manufacturer’s instructions. To visualize the effect of the enzyme, the capsules were labeled with DAPI and visualized by fluorescence microscopy. The experimental groups were divided into (I) empty capsules (control), (II) capsules with PEI + DNA, and (III) capsules with NaCl + DNA. The capsules evaluated were standardized at a concentration of 10^7^, with 250 ng/μL of internalized DNA (in the case of groups II and III). For analysis, microscopy was performed before and after enzymatic treatment.

### 2.12. Statistical Analysis

Graphs and statistical analyses were generated by GraphPad Prism version 9.0. For cytotoxicity assay and encapsulation efficiency analysis, One-way and Two-way ANOVA were applied, respectively, followed by Tukey’s post hoc tests. For thermostability evaluation, was employed the One-way ANOVA test followed by Šídák multiple comparison. Results with a *p*-value < 0.05 were considered statistically significant.

## 3. Results

### 3.1. Obtaining β-Glucan Capsules

**Figure 3:**
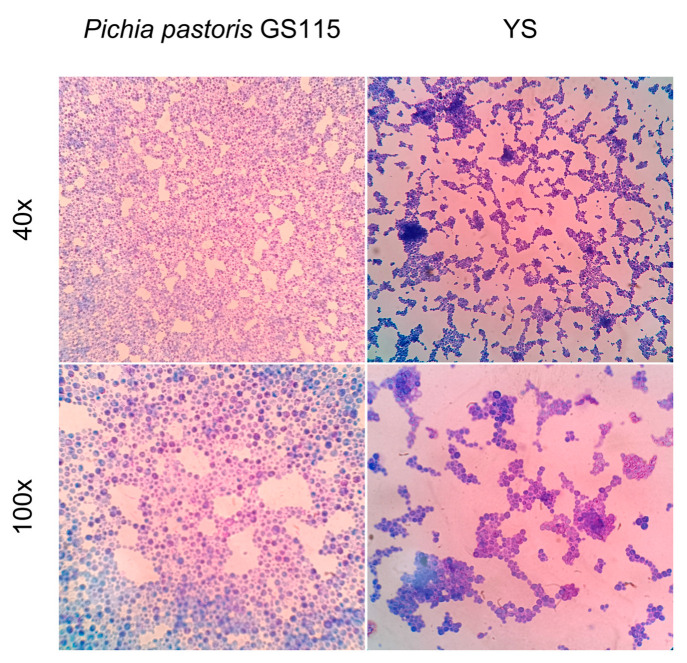
Optical microscopy of *Pichia pastoris* GS115 and yeast shells. Samples were stained with methylene blue and observed at 40× and 100× magnification. Microscopy with K55 OIT optical microscope (Kasvi, Pinhais, PR, Brazil) and image manual capture system.

Growth in culture medium for 48 h resulted in 18 g of wet yeast biomass obtained from 1 L of culture in liquid medium. After the YS preparation steps, the morphological changes between the whole wild yeasts and the capsules were analyzed by optical microscopy (Figure 3). The morphologies did not present relevant changes, demonstrating that the washes did not affect the cell wall architecture. After the treatments, the capsules formed clusters not observed in the whole yeast cells.

To prove that the capsules constitute a non-replicative delivery system, they were seeded on plates with a culture medium. It was possible to observe that there was no contamination in obtaining the capsules, and there was no reproductive activity. In contrast, the untreated *P. pastoris* GS115 exhibited the expected reproductive capacity, forming visible colonies in the culture medium (Supplementary Materials, Figure S1).

### 3.2. DNA Encapsulation Capacity by Yeast Capsules

**Figure 4:**
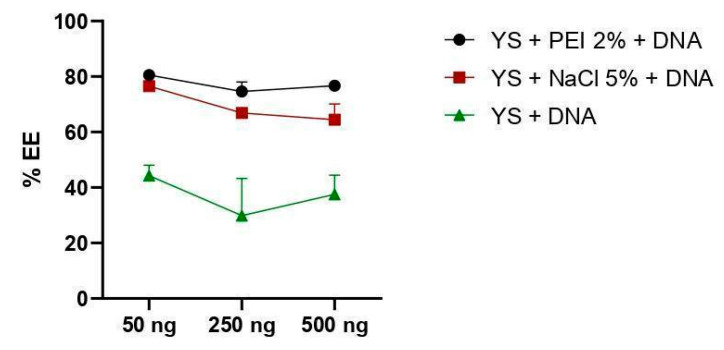
Comparison of the plasmid DNA incorporation efficiency between the three protocols adopted. %EE = encapsulation efficiency percentage. Different DNA concentrations were adopted to verify the loading capacity using 50, 250, and 500 ng/µL (x-axis). Values correspond to mean ± standard deviation.

The capacity to incorporate plasmid DNA was measured using a calculation to verify encapsulation efficiency (%EE). Capsules in which the genetic material was incorporated with PEI 2% showed a higher level of efficiency, regardless of the concentration of plasmid DNA initially used (50, 250, or 500 ng/µL), followed by the protocol using NaCl 5% and the formulation without ionic compounds. There was no significant difference between the use of PEI and NaCl, except for a concentration of 500 ng/μL, where DNA incorporation with the aid of PEI demonstrated greater efficiency (*p* < 0.0050). The absence of 2% PEI and 5% NaCl compounds decreased the encapsulation efficiency rate, regardless of the DNA concentration that was incorporated (*p* < 0.0001) (Figure 4).

### 3.3. Internalization of Plasmid DNA

**Figure 5:**
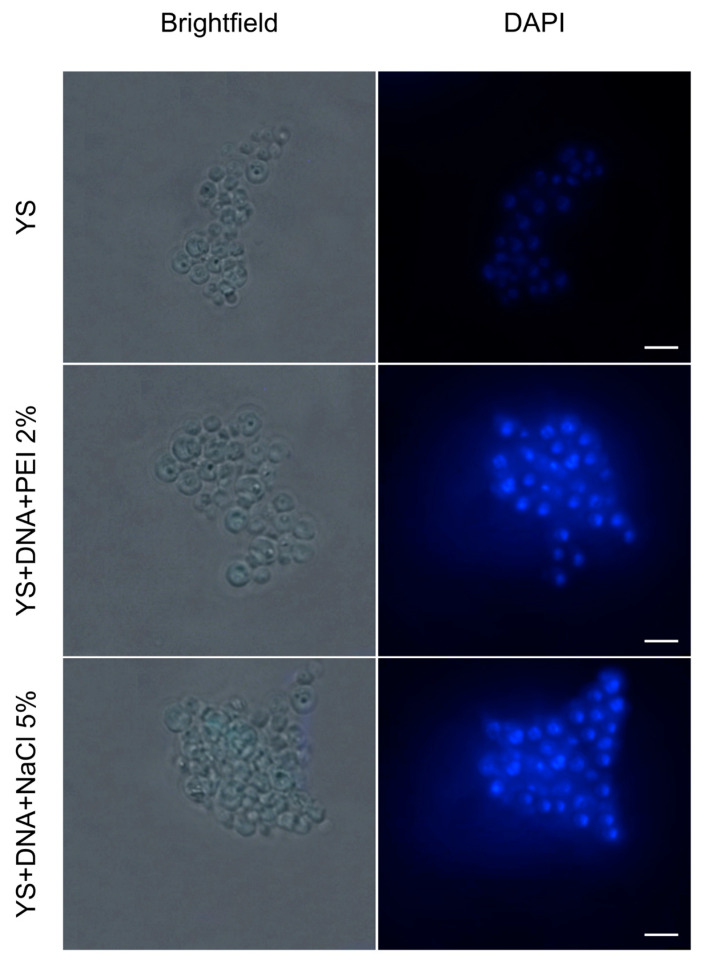
Fluorescence microscopy of capsules yeasts. YS: Empty capsules; YS + DNA + PEI 2%; and YS + DNA + NaCl 5%. Observation of the labeling of YS with DAPI (blue), indicating the presence of nucleic acid in the capsules. YS was used as a negative expression control and displayed only fluorescence background. Images captured through a Leica DMLB epi-fluorescence microscope (Leica, Wetzlar, Alemanha) at 100× magnification. Images were captured by the Leica DFC345 FX. Scale bar: 5 μm.

In Figure 5, we observe the control sample, corresponding to the empty YS, characterized by the absence of fluorescence due to the lack of genetic material. In contrast, in the YS prepared with the insertion of plasmid DNA, fluorescence was detected from the labeling with DAPI, evidencing the presence of genetic material and demonstrating the efficiency of the capsules in incorporating exogenous DNA.

### 3.4. Thermostability of Capsules and Retention of Plasmid DNA

**Figure 6:**
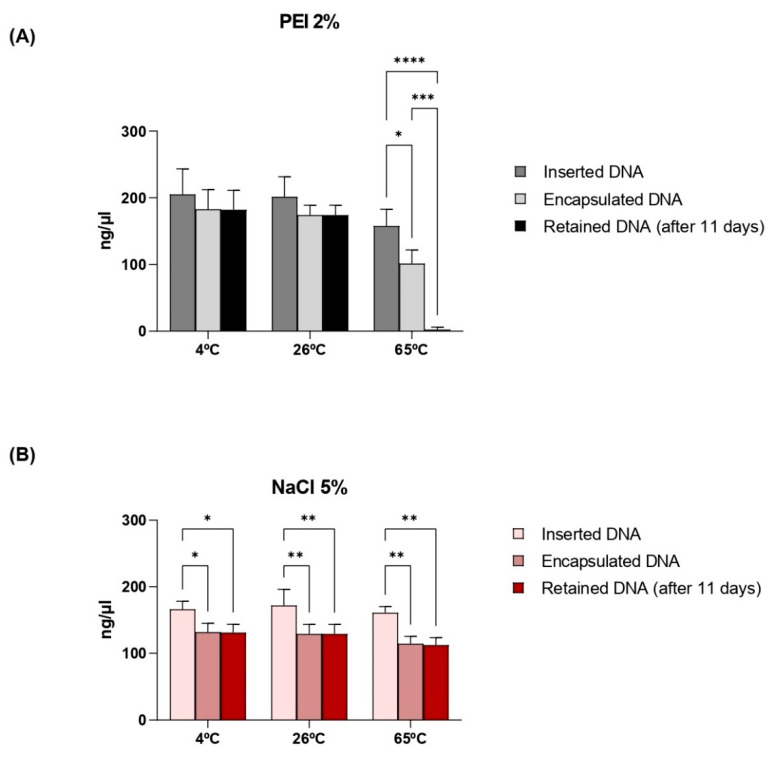
Evaluation of the maintenance of plasmid DNA inside the capsules under different heat treatments. (**A**) PEI 2% and (**B**) NaCl 5% were the compounds used to incorporate the DNA inside the capsules. The y-axis corresponds to the DNA concentration, and the x-axis corresponds to the temperatures that were tested. Bar = means ± SD. Asterisks represent statistical significance (* *p* < 0.05; ** *p* < 0.01; *** *p* < 0.001; **** *p* < 0.0001).

The ability to retain the incorporated DNA was evaluated using an 11-day interval between insertion and verification of loss. DNA retention at different temperatures was also evaluated in this study (Figure 6). Although it can be observed that not all of the DNA used for insertion is actually encapsulated, once inside the capsule there is retention of the material to be carried. Capsules whose plasmids were incorporated with the aid of NaCl 5% demonstrated stability in retention despite temperature variations. On the other hand, the material incorporated with PEI 2% is lost when incubating the capsules at 65 °C.

### 3.5. Phagocytosis of Capsules by Macrophages

**Figure 7:**
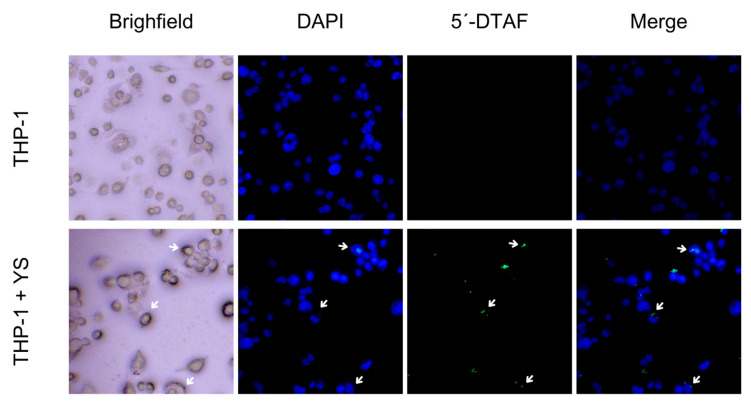
Phagocytosis verification by fluorescence microscopy. Groups: THP-1 cells only and THP-1 + YS (yeast shell), observed under bright-field and fluorescence microscopy. YS were labeled with 5′DTAF (green), and THP-1 cells were labeled with DAPI (blue). The white arrows highlight the YS phagocytosed by the macrophages. Images were captured through a fluorescence microscope (Motic AE31E) at 40× magnification. Images were captured by the Moticam S6 camera using Motic Images Plus 3.0 software. Scale bar: 5 μm.

The results of the phagocytosis assay are presented in Figure 7. In this figure, we observe macrophages (THP-1) without the addition of YS (negative control) and cells containing phagocytosed YS. The YS, labeled with a cell wall dye with affinity for β-glucans, were visualized by fluorescence microscopy. These results demonstrate the recognition and phagocytosis of YS by the macrophages.

### 3.6. Induction of Cytotoxicity

**Figure 8:**
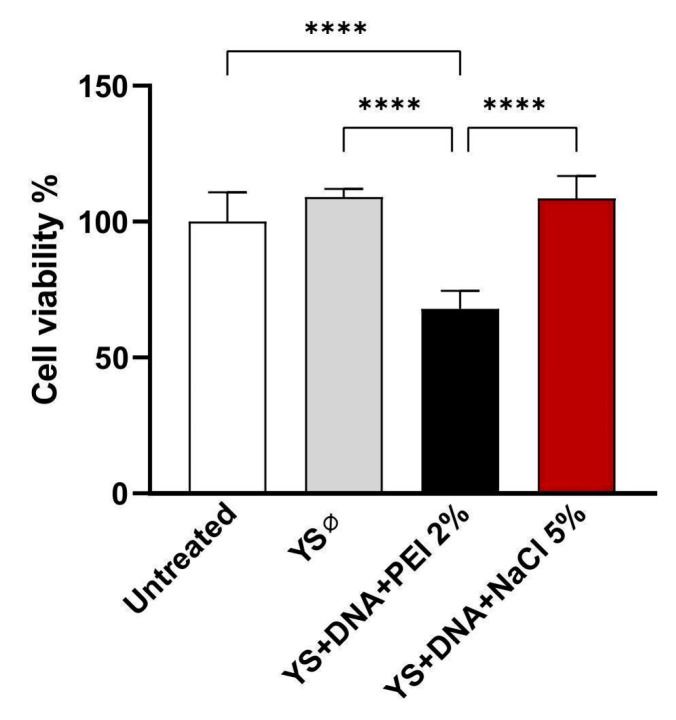
Cell viability evaluation by MTT assay. HEK 293-T cells were treated by incubation with yeast shells (capsules). Asterisks represent statistical significance (**** *p* < 0.0001), determined by the variance test (ANOVA). Values correspond to mean ± standard deviation.

The toxicity potential was assessed using the MTT assay after 24 h of exposure of HEK 293-T cells to the capsules, in a ratio of 2:1 (cells/capsules). It was noted that the preparations with 2% PEI in their composition led to a reduction in cell viability at the concentration tested, in contrast to the other experimental groups (Figure 8).

### 3.7. Zeta Potential (ζ) Analyses

According to the ζ results for empty capsules and DNA-loaded capsules, functionalization using PEI 2% and NaCl 5% proved effective, as evidenced by the variation in ζ between the control and the DNA-containing samples (Table 1).

The empty capsules treated with 2% PEI exhibited a slightly positive zeta potential (ζ = 2.8 ± 0.73 mV), indicating the interaction between PEI and the capsules, resulting in a slightly positive surface charge. This behavior is expected due to the cationic properties of PEI. When the capsules were loaded with pVAX through treatment with PEI 2%, a minimal variation in zeta potential was observed (ζ = 10.17 ± 0.17 mV). This result suggests that the DNA was efficiently incorporated and did not remain adsorbed on the surface, as the change in zeta potential remained consistent with values similar to those observed in the control.

On the other hand, the empty capsules treated with NaCl 5% exhibited a negative zeta potential (ζ = −4.23 ± 0.14 mV), indicating a predominantly negative surface charge, possibly due to the functional groups present on the yeast cell wall, such as β-glucans, without interference from the neutral charge of NaCl. The hypertonic NaCl solution, being electrolytic, may have partially neutralized these charges, reducing the magnitude of the negative potential. The capsules loaded with pVAX and treated with NaCl 5% showed a slightly less negative zeta potential (ζ = −3.18 ± 0.26 mV) compared to the empty capsules. This minimal variation suggests that the DNA was not exclusively retained on the surface.

### 3.8. Evaluation of Enzymatic Protection

**Figure 9:**
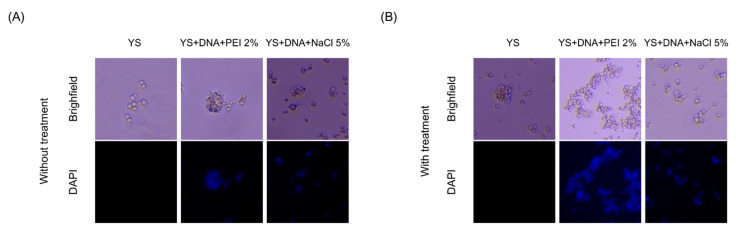
Comparison of DNAse assay results. (**A**) Empty yeast capsules and capsules with two insertion protocols (PEI 2% and NaCl 5%) whose inserted DNA (pVAX1) was stained with DAPI (blue color) without being subjected to DNAse treatment. (**B**) Empty yeast capsules and capsules with two insertion protocols with DNA stained with DAPI but subjected to DNAse treatment. It was possible to observe that there was no degradation of the DNA inserted in the capsules, even in medium containing DNAse. Images captured through a fluorescence microscope (Motic AE31E) at 40× magnification. Images were captured by the Moticam S6 camera using Motic Images Plus 3.0 software.

To verify the protection of the internalized plasmid against enzymatic degradation, a DNase assay was performed with microscopy analysis. As shown in Figure 9, there was no visible degradation of the internalized DNA, suggesting that the capsules protect the cargo from degradation and that this cargo is possibly inside the capsular structure and not on its surface since the fluorescence signal is similar in the pre- and post-treatment samples.

## 4. Discussion

Although several yeast species have been proposed as vaccine carriers and adjuvants, only *Saccharomyces cerevisiae* yeast has been applied to produce capsules derived from its cell wall. Despite the morphological similarities between the cell walls of *P. pastoris* and *S. cerevisiae*, there are some differences in the distribution of *P. pastoris* cell wall components, such as higher amounts of β-1,3-glucans, mannans, and chitin, which may increase the immunostimulatory potential concerning T lymphocyte activation [36]. Therefore, we propose a new vaccine delivery platform using β-glucan capsules derived from the *P. pastoris* cell wall.

Yeast cell wall extraction processes included washing steps with isopropyl alcohol [37]. These washes are necessary to remove lipids present in the cell wall, as well as to remove residual moisture and improve YS porosity [20]. However, it is also reported that alcohol can denature proteins, altering their three-dimensional structure [38]. Analyses by optical microscopy after these treatments showed similarities between the capsules and the intact yeasts, with oval structures and apparent preservation in the cell wall architecture (Figure 2). One characteristic that drew attention was the formation of clusters between the capsules that were not observed in the intact cells. This finding may be associated with the increased exposure of β-glucans caused by removing the mannoprotein layer from the cell wall of these yeasts and because these glucans have hydrophobic characteristics [39]. Increasing the hydrophobicity levels of polymers used as delivery systems decreases the release rate and degradation of the polymer matrix due to the increased affinity between polymer and payload [40,41].

In this study, the pVAX1 vector was used as a model plasmid to evaluate the capsules for incorporation and retention of a DNA vaccine. This vector has 3 kb and eukaryotic sequences necessary for expression in mammalian cells to minimize the possibility of chromosomal integration [42]. For the insertion of plasmid DNA into the capsules, three protocols were tested. The first employed a polymer that interacts electrostatically with the capsules and the inserted DNA (PEI 2%), the second using an electrolytic solution (NaCl 5%), and the third without the addition of solutions that have affinity to charges and that interact with the capsules and DNA (PBS). It has been proposed that the porosity of YS can cause leakage of the payload. Therefore, it may be interesting to use polymers that interact electrostatically, such as PEI, forming a protective matrix bypassing these limitations [43].

On the other hand, NaCl, as an electrolyte, provides Na⁺ and Cl⁻ ions that can influence the charge distribution on the surface of yeast capsules and DNA. These ions may reduce the intensity of the negative charges present on these molecules, altering electrostatic interactions and potentially contributing to the partial neutralization of these charges. This neutralization can reduce the electrostatic repulsion between the DNA molecules and the capsule surface, facilitating the approach and internalization of the DNA [44]. In this way, NaCl contributes to creating an ionically balanced environment, which favors the formation of interactions between the DNA and the capsules. However, complete charge neutralization may also be influenced by other factors, such as pH or the presence of additional ions, which play a role in modulating these interactions. Thus, further studies are required to provide a more comprehensive understanding of the mechanism.

The use of NaCl facilitates capsule permeabilization through plasmolysis, creating a significant osmotic gradient between the external environment and the capsule interior. This process enables the diffusion of compounds into the capsule [15]. Several compounds have been incorporated into the capsules following this methodology, such as curcumin, Antarctic krill oil, and chlorogenic acid [32,33,45]. Ours is one of the pioneering works in employing NaCl for plasmid DNA encapsulation, demonstrating its potential use.

The DNA encapsulation efficiency (EE%) was calculated according to the model described by Yu et al. (2015), relating the payload to the difference between the load inserted and the load not absorbed by the capsules [46]. As observed in Figure 4, the capsules formulated with PEI 2% polymer reached above 80% efficiency at different concentrations of inserted DNA, similar to Soto et al. (2008) [14]. Among the advantages of these polymers, PEI is one of the best cationic polymers for forming nano-complexes with DNA. However, as previously described [47], this compound induces significant cytotoxicity, as illustrated in Figure 7. In contrast, there was no significant change in the viability of eukaryotic cells (HEK 293-T) incubated with capsules with DNA incorporated with NaCl 5%.

Capsules formulated with NaCl 5% showed an EE higher than 60% (Figure 4). These findings demonstrate greater efficiency compared to other studies that used similar methodologies. Paramera et al. (2011) reported that microcapsules containing curcumin achieved an EE% of around 35% [32]. However, the difference in encapsulation efficiency may be related to the nature of the material inserted into the capsules.

In the incorporation protocol that did not employ electrostatic interaction agents, the efficiency was less than 50% at the different DNA concentrations, especially regarding the insertion concentration of 500 ng/µL, which presented an EE% below 3%. Negatively charged materials, such as DNA, tend to adhere more easily to positively charged surfaces compared to uncharged or negatively charged materials [48,49,50,51]. This finding highlights the importance of stabilizing agents, such as PEI or NaCl, for effective DNA retention. The absence of these polymers results in rapid release of the payload, indicating that this protocol is not suitable for applications requiring prolonged DNA retention.

The selected formulations, composed of NaCl 5% and PEI 2%, were freeze-dried and verified for their thermal capacity to understand the behavior of YS in retaining this DNA at extreme temperatures. After 11 days, there were minimal losses of inserted DNA at 8 °C, 26 °C, and 65 °C for the protocol with NaCl 5%. On the other hand, the protocol with 2% PEI could not retain the DNA efficiently, resulting in a loss of over 93% of the material when subjected to 65 °C. Although the PEI protocol demonstrated a higher EE%, the protocol with NaCl stood out for its better payload retention, especially under high-temperature conditions. According to Paramera et al. (2011), YS associated with NaCl 5% favors their encapsulation at temperatures above 35 °C, increasing the EE% values by at least two-fold [32].

Fluorescence microscopy was used to visualize the DNA inside the capsules. After the insertion of the plasmid constructs, the fluorescence signal was detected inside the YS, demonstrating the internalization of the genetic material. The DNA delivery system by yeast capsules had already been demonstrated, but only for structures derived from the wall of *S. cerevisiae* [14,52]. It is also worth noting that the internalized material, by both protocols, is protected from enzymatic degradation, as observed in the test performed with DNase. These data are fundamental and should be further investigated because they indicate the functionality of this delivery system in protecting the encapsulated cargo.

The phagocytic process of yeast involves interaction with receptors such as the mannose receptor in macrophages, the β-glucan receptor, and the complement receptor type 3, responsible for recognizing non-opsonized yeast particles [53]. Therefore, an in vitro assay was performed to verify whether macrophages can phagocytose *P. pastoris* capsules, as already described for capsules obtained from *S. cerevisiae* [12,18].

According to Carneiro et al. (2014), most studies focus on the interaction between phagocytes and live yeast cells [54]. However, inactivated cells can also stimulate immune responses since the cell wall PAMPs can be identified in both live and inactivated cells [54]. Thus, despite the structural modifications resulting from the treatments applied to produce yeast capsules, they can be internalized by APCs. However, the mechanisms and parameters of yeast capsule phagocytosis still need to be better elucidated.

The zeta potential of the capsules was analyzed under different conditions to evaluate the effect of PEI or NaCl treatment on the modification of the surface charge and DNA incorporation (pVAX). The minimal variations between the control and the samples containing plasmid indicated that the DNA was not adsorbed on the surface, thus being incorporated inside the capsules. Regarding the final charges of the YS, the capsules treated with PEI presented a positive surface charge, while those treated with NaCl maintained a negative charge, although close to zero.

Previous studies have reported that cationic polymers and DNA complexes with positive surface charge are suitable for efficient endocytosis and gene delivery [55,56,57]. This behavior can be attributed to the favorable electrostatic interaction between the positive charge of the capsules and the cell membrane, which is negatively charged. This principle is consistent with the results obtained for PEI-functionalized capsules, suggesting that these capsules are more likely to facilitate cell DNA internalization.

On the other hand, NaCl treatment, which resulted in negatively charged capsules, may reduce the interaction with cell membranes, decreasing the internalization efficiency. It has been shown that particles with negative zeta potential exhibit lower interaction with negatively charged cell membranes [58]. In contrast, as observed by Nguyen et al. (2009) studying the CMC compound (P(68)-10 CMC 2) in MTT assays, approximately 80% of the cells remained intact at the highest polymer concentration. In this study, it was suggested that this aspect occurred due to the negative zeta potential, which showed less interaction with the negatively charged cell membrane. Furthermore, the other tested nanocomposite formulations with positive surface charges exhibited higher toxicity profiles [59]. These results reinforce the importance of considering not only zeta potential but also the balance between surface charge and cytotoxicity when designing efficient and safe delivery systems.

Thus, this study demonstrates that YS in different formulations using ionic polymers internalize a large concentration of DNA and contain this material at distinct temperatures. In addition, the *P. pastoris* capsules are recognized and phagocytosed by macrophages, becoming a promising vaccine delivery system. Therefore, more comprehensive studies are needed to make this methodology viable.

## 5. Conclusions

The results obtained in this study innovatively reveal the development of a vaccine delivery platform based on the use of capsules derived from the *Pichia pastoris* cell wall. The YS showed efficiency in encapsulating DNA through different protocols applied by electrostatic interactions with 5% NaCl and 2% PEI, resulting in encapsulation efficiency (EE%) above 63% and 80%, respectively. Although the PEI protocol demonstrated better encapsulation efficiency, it reduced cell viability at the concentration tested. Furthermore, the protocol using 5% NaCl showed higher thermostability, retaining the inserted DNA even at extreme temperatures such as 65 °C. These findings suggest it is the more suitable methodology for DNA incorporation into the capsules. The retention of DNA for more than ten days at room temperature also reveals an advantage of yeast capsules as a delivery system, considering a storage that does not require refrigeration chains. Phagocytosis assays demonstrated recognition and internalization of YS by macrophages, representing a promising vaccine delivery for antigen-presenting cells. Further studies are needed on the characterization of this approach, especially regarding this system as an immunomodulator. Furthermore, as DNA vaccines are carried within these β-glucan capsules, it will be needed to evaluate the assessment of DNA release in target cells and subsequent expression of the gene inserted in this molecule, which is considered the vaccine antigen.

## Figures and Tables

**Table 1:** Zeta potential (ζ) values (mean ± SD ^1^) of empty capsules and capsules with DNA after treatment with PEI 2% and NaCl 5%.

Sample	ζ (mV)
Empty capsule PEI 2%	12.8 ± 0.73
Capsule pVAX1 PEI 2%	10.17 ± 0.17
Empty capsule NaCl 5%	−4.23 ± 0.14
Capsule pVAX1 NaCl 5%	−3.18 ± 0.26

^1^ SD: standard deviation. The ζ measurements were conducted in triplicate.

## Data Availability

All relevant data from this study are available from the corresponding author.

## Supplementary Materials

The following supporting information can be downloaded at: https://www.mdpi.com/article/10.3390/vaccines12121428/s1, Figure S1: Verification of YS metabolic activity.
