# Supplementary material for: Pichia pastoris-Derived β-Glucan Capsules as a Delivery System for DNA Vaccines
Source: Vaccines (Basel). 2024 Dec 18;12(12):1428. doi: 10.3390/vaccines12121428 (PMC11728682; doi:10.3390/vaccines12121428)
Supplement: Supplementary file 1 [file vaccines-12-01428-s001.zip › vaccines-3318775-supplementary.pdf]

Supplementary Materials

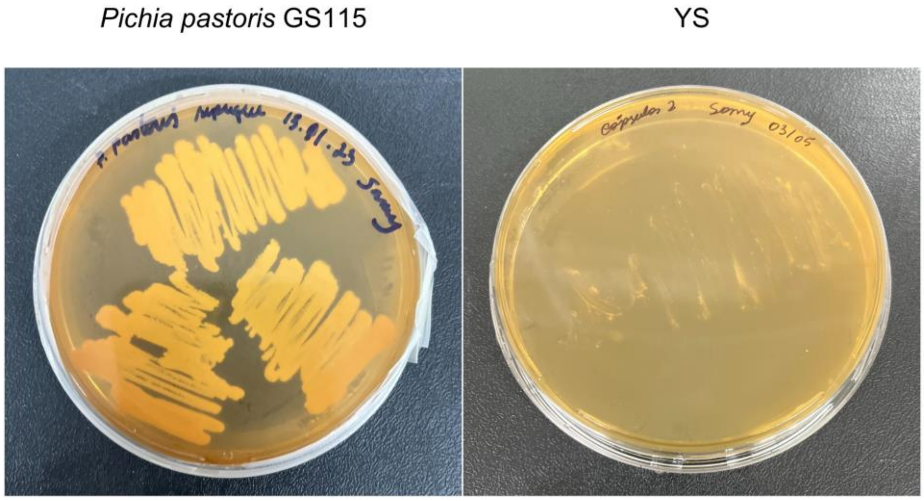

Figure S1. Verification of YS metabolic activity. In the image on the left, the positive control with *P. pastoris* GS115 shows microorganism growth. On the right, the yeast shells (YS) exhibit no metabolic activity. The plates contained solid YPD and were incubated for 48 hours at 30°C.
